# Interprofessional Competency Frameworks in Education

**DOI:** 10.15694/mep.2019.000056.1

**Published:** 2019-03-15

**Authors:** Andrea Smilski, Mary Parrott

**Affiliations:** 1 College of the North Atlantic - Qatar

**Keywords:** Interprofessional collaboration, Competencies, Interprofessional education, Health education, Canada, Qatar

## Abstract

This article was migrated. The article was marked as recommended.

Interest in Interprofessional collaboration (IPC) in health care is increasing, as concerns about patient safety, resource shortages, and effective and efficient care have become explicit priorities. Although there are many exemplars of Interprofessional education (IPE) for collaborative, patient-centered care, there is little in the literature to describe competencies for an Interprofessional collaborative practitioner.

Although there are many perspectives on the concept of Interprofessional collaboration, there is scarce literature on the subject related to its application in health education programs. This article describes two Interprofessional competency frameworks that have been developed in Canada and Qatar. These particular frameworks are highlighted because of College of the North Atlantic’s (CNA-Q) tie to Canada as a Canadian College operating within Qatar. The frameworks, which have been respectively applied within their own contexts, offer opportunities for the application of Interprofessional competencies elsewhere in the worldwide. The models proposed are reviewed and their utility for educators and practitioners is discussed.

The first framework is a Canadian competency framework for IPC that: (1) considers descriptions of collaborative practice and (2) uses existing literature to support a model for describing competencies for collaborative practice. The second framework of Interprofessional health competencies developed in Doha, Qatar originated from a National Priorities Research Project supported by the Qatar National Research Fund. It builds upon a model developed by Qatar University (QU) ( El-Awaisi *et al.,* 2017) and the Canadian National Interprofessional Competency Framework for Collaborative Practice ( Johnson, *et al.,* 2015). It provides guidance for implementation of IPE in pre- and post-licensure settings.

## Introduction

Increasingly Interprofessional collaboration (IPC) is recognized as a means of improving patient outcomes and fiscal effectiveness of care in a variety of settings that may include primary health care, acute care, rehabilitation, and community care ( McPherson, 2001). Health professionals must be able to work collaboratively within Interprofessional teams or groups in order to ensure consistent, continuous, safe, and reliable care. Complex health care environments faced with issues related to patient safety, human resource shortages, and increasing health care needs compound the challenge of IPC.

Policy makers from Canada, the United Kingdom, New Zealand, and the Unites States are recommending changes in health professional curricula to instill student acquisition of competencies that facilitate collaborative practice ( McPherson, 2001; Response to the Romanow Report, 2002). Through IPE, students and practitioners can develop competencies in the form of knowledge, skills, attitudes, and behaviors that will enable them to work collaboratively throughout their chosen careers. To achieve the goal of improved health outcomes, it is important to define the essential competencies required for collaborative practice. It is also important to develop and deliver educational interventions to ensure their adoption.

When educators across departments and among service delivery institutions share common models and frameworks, their approaches to introducing new content within health professional education are more consistent ( Harden, 2002). Common frameworks, such as those associated with Interprofessional education, help educators to: plan curriculum and learning strategies; allocate resources for instruction; develop a sense of commitment to the proposed implementation process; and legitimize unfamiliar curricular content and methods. The process of introducing Interprofessional health education is similar across all health and human service education programs.

For the purposes of this article IPE is education that supports the development of two broad concepts in learners: Collaborative Competence ( Meads *et al.,*2005), and; Professional Competence ( Barr, 1998). Collaboration involves different degrees of proximity in time or space and different levels of complexity. It may be co-located and concurrent, (i.e., people working together on the same task at the same time in the same place). It may be sequential, (i.e., a series of steps to provide seamless care). Lastly, it may be virtual (i.e., researchers working with practitioners/organizations to improve services, specialist teams holding teleconferences with patients in the same location or in different locations). According to Barr (1998), Collaborative competencies include several activities such as: communication/networking; understanding roles; respecting /valuing other professions; managing change and conflict; working together; developing, supporting, and accepting each other, and; facilitating teamwork. Professional competencies relate to scope of practice (requisite skills, knowledge and abilities), professional standards, professional boundaries, self-regulation, and ethical conduct. Barr (1998) distinguishes among three main professional competencies that all provide rational for joint training of students. These are common, complementary, and collaborative. Common Competencies are those held in common between all professions. Complementary Competencies are those that distinguish one profession from another. Collaborative Competencies are those necessary to work effectively with others and evidence suggests that joint training develops them. When these two broad areas are combined into “Collaborative Professional Competencies”, they are foundational to Interprofessional competence.

This paper begins with support for the adoption of an Interprofessional competency framework. Competencies are described and the literature supporting the development of competency frameworks is highlighted. The knowledge, skills, and attitudes for collaborative behaviors are offered, and the Canadian Interprofessional competency framework is described ( Canadian Interprofessional Health Collaborative, 2009). Lastly, the Interprofessional competency framework initiatives and developments in Qatar are described and their relationship to the Canadian Interprofessional Health Collaborative (CIHC) framework is highlighted. In the conclusion, a discussion about the application of the competency framework to learning outcomes and assessment of competency acquisition is provided.

## In Support of an Interprofessional Competency Model

Professional health education programs traditionally use markers or indicators to determine when a student has achieved a required level of proficiency in knowledge, skills, and professional attitudes for entry to professional practice. These indicators are profession specific and quite prescriptive. The profession specific regulators, educators, and practitioners identify and guide competencies within their profession’s education programs. This focus can limit attention as to how these same professionals interact with members of other health professions. Generic mentions of “teams” and “communication” within profession-specific competency frameworks fail to present accurate pictures of Interprofessional collaboration. The limited literature on Interprofessional education suggests an absence of a commonly agreed upon Interprofessional competency framework ( Barr, 1998; Suter, 2009).

The CIHC is the national Canadian organization for activities related to Interprofessional education, collaboration in health care practice, and patient-centered care. Their scope of interest bridges health, education, and the professions. The role of CIHC is to discover and share promising practices that promote Interprofessional education and collaboration in order to enhance patient care. In 2007, with funding from Health Canada, the CIHC Interprofessional Competency Working Group began their work to develop a national Interprofessional competency framework in Canada.

## Literature Review of the Pan-Canadian and Qatar Frameworks

The CIHC Interprofessional Competency Working Group reviewed the literature on competencies and existing regional competency frameworks for Interprofessional education and collaboration ( Canadian Interprofessional Health Collaborative, 2009). The working group conducted a search for articles related to general and Interprofessional competencies using search terms such as post-secondary, higher education, competence theory, competency-based education, collaborative learning, and collaborative practice.

In Canada, from 2005 to 2008, each region developed Interprofessional competency documents to meet local needs. CIHC (2009) identified that different foundational perspectives and approaches to competence shaped these documents. Despite this, the working group found that common competencies did exist and these included: patient-centered care; collaborative working relationships; teamwork; Interprofessional communication (incorporating listening, negotiating, consulting, interaction, discussion/debate, and attending to nonverbal parameters); shared leadership; self-awareness (reflection); and evaluation ( Canadian Interprofessional Health Collaborative, 2009). These common elements formed the basis for the pan-Canadian framework.

A review of the literature of IPE in Qatar reveals a history of collaboration between post-secondary medical/health educational institutions and hospitals. Since many of the institutions were new to the region in the early 2000’s, a pioneering spirit fueled with passion for education and practice created a unique development opportunity. Collaboration largely centered around two main themes: a review and debate about health education in Qatar, and; development of a relevant IPE model for the State of Qatar and the region. In 2002, Weil Cornell Medical Center - Qatar (WCMC-Q) and the CNA-Q began programs for health care education of pre-med students and allied health professionals. QU accepted pharmacy students to their pharmacy program in 2006. The University of Calgary-Qatar (UC-Q) began its first Bachelor of Science in Nursing (BSN) program in 2007. These educational institutes were joined by Hamad Medical Center and Sidra Hospital, and, in 2009, they collectively formed the Qatar Interprofessional Health Council (QIHC). QIHC’s mission is to work with stakeholders within Qatar, the Middle East region, and internationally to support collaborative IPE initiatives in both education and professional practice ( Johnson, *et. al.,* 2011).

Guided by Canadian accreditation standards for the College of Pharmacy program, QU developed a framework that supported IPE within an undergraduate, pre-licensure setting. They applied Miller’s Pyramid of Clinical Competence ( Miller, 1990) that guides development and delivery of learning to support students as they move from knowing to demonstrating and then to applying learning. Along with Miller’s Pyramid of Clinical Competence, QU adopted a model outlined by the University of British Columbia (UBC), to guide content, level of skill complexity, and delivery methods ( Charles, Bainbridge, and Gilbert, 2010). IPE activities were developed for students from pharmacy, nursing, physician and allied health professions. The shared domains of IPE competency include professional role clarification, Interprofessional communication, patient-centered care and shared decision-making ( El-Awaisi *et al.,* 2017).

Johnson et al. (2015) reviewed and researched the framework developed by QU’s College of Pharmacy, as well as the Accreditation Council for Graduate Medical Education (ACGME) from the United States, the CIHC, and the Interprofessional Core Competency Framework (ICCF), both from Canada. One of the outcomes of the research was the development of the Core IPE Health Competencies, which is a set of core Interprofessional competencies applicable for post-secondary learners and licensed practitioners to serve local needs ( Johnson *et al.,* 2015). This model recognizes three general categories that align with Barr’s findings in 1998. The categories include discipline specific competencies, common competencies, and core IPE competencies. The four IPE domains identified are role clarification, patient- centered care, shared decision-making and Interprofessional communication.

Since the development of the QIHC there has been ongoing research, workshops, and simulation events in Qatar related to Interprofessional competency attainment. In a qualitative study on IPE, Wilbur and Kelly (2015), found that students from Qatar’s nursing and pharmacy programs who collaborated on IPE learning, identified value and importance in participating in IPE activities. They indicated that IPE contributed to competency learning and improved professional practice and patient outcomes.

IPE events that include several different post-secondary institutions in Qatar provide an opportunity for students to problem solve in interdisciplinary teams. A recent IPE event brought together 94 learners from post-secondary colleges and universities in Qatar to learn about IPE though interdisciplinary group work. The event promoted the IPE skills of teamwork and communication. Students had the opportunity to learn about other health care professions work, the positive impact cooperation could have on patient care, and the value of IPE to effective care ( Qatar University, 2017).

## Development of a National Canadian Competency Framework for Interprofessional Collaboration

During the development of a nationwide Interprofessional competency framework the CIHC Interprofessional Competency Working Group focused on Interprofessional collaboration since this was the main objective. The knowledge, skills, attitudes, and behaviors required to produce Interprofessional collaboration were of particular interest. Based on this, several assumptions derived primarily from Roegiers ( Roegiers, 2007) and Tardif ( Tardif, 1999), created the basis for the competency framework. These include:

• Competency statements are strong over-arching statements that last over a long time.
• Competency descriptors identify specific knowledge, skills, attitudes, values, and judgments that are dynamic, developmental, and evolutionary.
• Interprofessional learning is additive and reflects a continuum of learning.
• Interprofessional collaborative practice is essential for improvement in patient/client and family health outcomes.
• The level of Interprofessional competence is dependent on the depth and breadth of opportunities for education and practice with, from, and about other disciplines.
• Adoption of Interprofessional competencies into health care professional education programs will occur at different rates depending upon the level of learner/practitioner and the complexity of learning tasks.
• Adoption of Interprofessional competencies may require a shift in how learners, educators, and practice environments conceptualize collaboration.

Although competency statements typically describe generic end-points or desired outcomes, the associated dialogues are individualized and flexible based on a given learner or practitioner experience, as well as their learning or practice context. Competency descriptors facilitate curriculum development that build specific skills, knowledge, and attitudes over time. Therefore, they have an additive quality as the complexity of learning and practice experience increases. By integrating the competencies into a framework, learning experiences can become evolutionary in nature, allowing learners and practitioners to achieve competencies for collaborative practice in different ways, at different levels, and in different contexts. It represents a part of the lifelong continuum of Interprofessional learning at pre- and post-licensure levels and supports curricular development and performance assessment along this continuum.

## The Canadian Interprofessional Competency Framework

**Figure 1.**
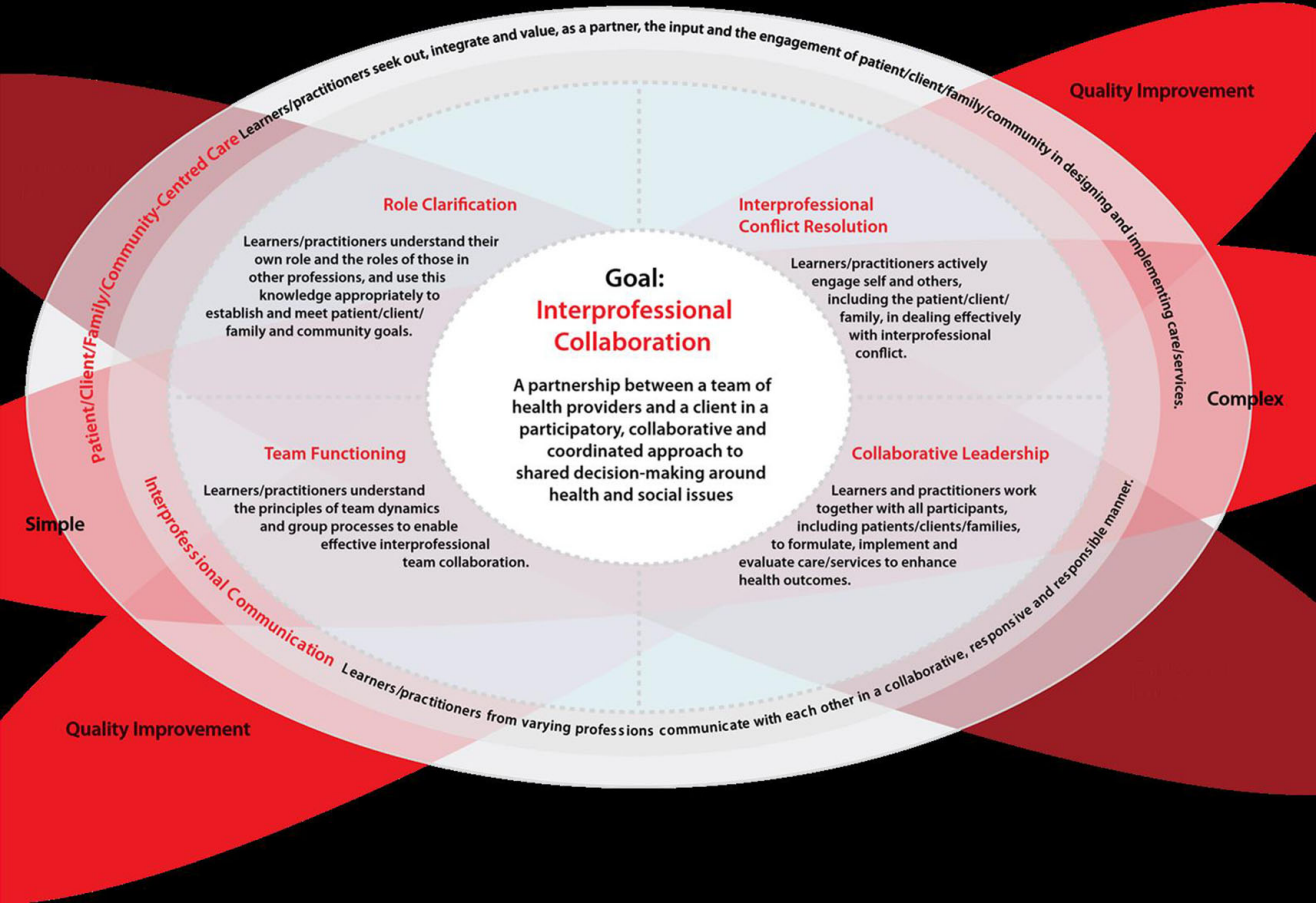
The CIHC National Competency Framework for Interprofessional Collaboration, 2009

The National Interprofessional Competency Framework is an evolving concept that will continue to change over time as educators, practitioners, and researchers become more familiar with the domains and descriptors. Currently, the framework presents 6 interconnecting Interprofessional competency domains - role clarification, patient/client/family/community-centered care, team functioning, collaborative leadership, Interprofessional communication, and dealing with Interprofessional conflict - all of which are essential to demonstrate Interprofessional collaboration ( Figure 1). None of the domains supersede the others and all are required to achieve Interprofessional collaboration.

Although all 6 domains contribute to the goal of Interprofessional collaboration, patient-centered care and Interprofessional communication are elements that influence the other 4 domains. This is depicted in Figure 1 as they are located on the outer circle to envelope the other domains. Within each domain is a competency statement with several associated descriptors. In addition to the domains, there are 3 background areas for consideration that broadly influence the interpretation and application of the competency framework. These are: simple to complex; context, and; quality improvement. The skill level in Interprofessional collaboration required along these continuums will differ in each situation. Simple encounters may be addressed adequately by 1 or 2 health care providers, whereas more complex cases may require a large team of providers, thus increasing the complexity of the collaboration. Context will also influence the application of the competency framework. In specific areas of practice such as mental health, geriatrics, and pediatrics, well-established, large teams allow an Interprofessional team to consolidate their collaborative practice approach over time. In an emergency unit the encounters might be short and the players might change frequently, requiring a different, although equally important, form of collaboration. Practitioners likely will use core collaborative practice skills when changing to a new context or practice until they develop a new skill set specific to the new context. Patient safety guides Interprofessional education, and therefore, a third consideration underpinning the competency framework is quality improvement. The specific domains and competency statements are explained below (CIHC, 2009).

Sourced from: Canadian Interprofessional Health Collaborative (CIHC, 2009)

**1. Domain: Role Clarification** ***Competency Statement:*** Learners/practitioners understand their own role and the roles of those in other professions, and use this knowledge appropriately to establish and meet patient/client/family and community goals.

**2. Domain: Patient/Client/Family/ Community-Centered** ***Competency Statement:*** Learners/practitioners seek out, integrate, and value, as a partner, the input, and the engagement of patient/ client/family/community in designing and implementing care/services.

**3. Domain: Team Functioning** ***Competency Statement:*** Learners/practitioners understand the principles of team dynamics and group processes to enable effective Interprofessional team collaboration.

**4. Domain: Collaborative Leadership** ***Competency Statement:*** Learners/practitioners understand and can apply leadership principles that support a collaborative practice model.

**5. Domain: Interprofessional Communication** ***Competency Statement:*** Learners/practitioners from varying professions communicate with each other in a collaborative, responsive, and responsible manner.

**6. Domain: Dealing with Interprofessional Conflict** ***Competency Statement:*** Learners/practitioners actively engage self and others, including the client/patient/family, in positively and constructively addressing Interprofessional conflict as it arises.

As mentioned, while competency statements do not change, descriptors are flexible and change depending on the level of the learner or practitioner or on the context of practice. It is important to remember that 3 concepts, the context of practice, the complexity of the encounter or situation, and the overarching philosophy of quality improvement, underpin the 6 domains.

## Development of a National Qatar Competency Framework for Interprofessional Collaboration

The first meeting of the QIHC was held in June 2009. At this time their goal was to bring together health care educators and health care practitioners from a variety of health professions to discuss health education and to promote IPE within Qatar and the region ( Johnson, 2011). The QIHC rolled out a series of panel discussions and open town-hall forums to introduce IPE to health care students, educators and administrative staff at QU, UC-Q, CNA-Q and WCMC-Q. Since then, these colleges and universities continue to bring their students together for events where students engage in IPE, problem-based learning, and learn about their own profession, others’ professions, and the role of interdisciplinary teams ( Qatar University *,* 2017).

In January 2013, driven by the recommendations of the Canadian Council for Accreditation of Pharmacy Programs (CCAPP), QU established an IPE committee (IPEC) to guide the development and implementation of IPE competencies for the College of Pharmacy program. The team had representation by faculty from QU, UC-Q, CNA-Q and WCMC-Q. Consistent with the UBC model, QU adopted the idea that there are “optimal learning times” that depend on a learner’s level of learning and stage of readiness ( Charles, Bainbridge, & Gilbert, 2010). This provided a platform for assessment of competence based on levels ranging from exposure, to immersion, and then mastery. They developed a successful model to guide the implementation of IPE into pre-licensure health care curriculum ( El-Awaisi et al., 2017).

**Figure 2.**
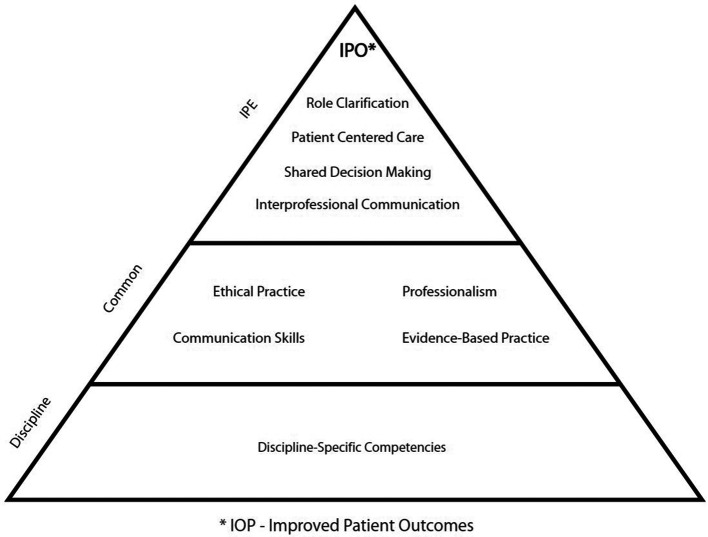
Core IPE Health Competencies Model Developed for Qatar, Johnson et al. 2015

In a research project to develop a culturally relevant set of core competencies that would apply to both learning and practice environments, a team of deans and directors from colleges, universities, and hospitals in Qatar reviewed competency literature from several sources. These include: the ACGME, United States; the CIHC and ICCF, Canada; and, the framework from the College of Pharmacy QU. The multidisciplinary team distilled the information into four core domains, and developed the Core IPE Health Competencies (see Figure 2). These include: role clarification; patient centered care; shared decision -making; and, Interprofessional communication. The model is applicable to both pre- and post- licensure setting and has been successfully applied in many workshops with clinical scenarios locally ( Johnson *et al.* 2015).

The Interprofessional Education Student Association - Qatar, founded in 2014, provides an opportunity for health care students in Qatar to learn about IPC together in annual events (Qatar: IPE Student Association Organizes Its Third Annual IPE Forum , 2018). The 4th annual IPE forum was held in January 2019 with participants from CNA-Q, UC-Q, QU, and WCMC-Q. Students from diverse disciplines including medicine, nursing, nutrition, pharmacy, allied health, public health and sports medicine attended. It provided students the opportunity to hear from keynote speakers and participate in judged competitions in IPE framework competencies.

### 1. Domain: Role clarification

**Definition:** Health care students and professionals understand and respect the role and responsibility of all stakeholders. [2]

Relevant stakeholders were identified as students, professionals, patients, and family.

*Competencies:*

Role:

1. Demonstrates through application an understanding of their own role. [2]
2. Understands the scope of professional practices and roles of each member of the health care team. [3]

General:

1. Demonstrates respect for other health care professionals’ roles and responsibilities. [2]

### 2. Domain: Inter-professional communication

**Definition:** Health care students and professionals communicate in a collaborative, responsible, and culturally sensitive manner. [2]

Competencies:

Patients:

1. Utilize effective communication skills with the patients and their family members. [1, 3]
2. Disclose and effectively communicate ethical issues with the patients and their family members. [3]
3. Demonstrate through application an understanding of respect, empathy, and cultural sensitivity when communicating with the patients and their family members. [4]

Health care Professionals:

1. Demonstrate through application an understanding of the principles of teamwork communication. [2]

General:

1. Communicate to ensure common understanding of health care decisions. [2]
2. Ensure that accurate and timely information reaches those who need the information. [3]
3. Understand and apply to the organizations (health agencies) approved standards of communication, internally and externally. [3]

### 3. Domain: Patient centered care

**Definition:** Health care students and professionals seek out, integrate and value the input and the engagement of the patient and family as part of the health care team [Adapted from CIHC]

Competencies:

Health care Professionals:

• Create and sustain a therapeutic and ethically sound relationship with the patients and their family members. [1]
• Demonstrate caring and respectful behaviors when interacting with the patients and their family members. [1]
• Performs professional roles and responsibilities in a culturally respectful way. [2]

General:

• Advocate for quality patient care and assist patients in dealing with health care system complexities. [1]
• Provide education and support to the patients and their family members in a respectful and understandable manner. [1; 2; 4]
• Encourage discussion and enable the patients and their family members to make informed choices about their health care. [1; 2; 4]
• Include patients and their family members as part of the health care team.

### 4. Domain: Shared decision-making

**Definition:** Health care students and professionals include all stakeholders in the decision-making process regarding patient health care outcomes.

Competencies:

Health Professionals:

• Exchange knowledge and skills with other members of health care teams at all times to promote collaborative practice when assessing, developing, and planning during the patient care processes. [2; 3; 4]
• Acknowledge each discipline’s perspective during team meetings and, or inter-professional exchanges during the patient care process. [3]
• Involve all members of the team as well as the patient and their family members in the decision-making process related to planning and implementing care. [3]

General:

• Actively seek to create and support a climate of shared decision-making and collaborative practice. [2]
• Facilitate the integration of evidence-based practice into the shared decision-making process. [3]

[1] ACGME

[2] CIHC

[3] Interprofessional Core Competency Framework

[4] College of Pharmacy

( Johnson et al. 2015)

## Application

Understanding what shapes practice allows curriculum developers to build learning frameworks that incorporate learners and practitioners’ competencies for success. It also helps educators set clear learning goals aimed at training competent collaborative practitioners. The learning framework draws upon different levels of learning as learners and practitioners move from simple to complex activities and from one practice context to another. Competencies are the foundation upon which assessment of ability can be built, but they do not describe the levels at which individuals are expected to perform. One competency statement cannot stand-alone, and therefore, the competency framework represents an integrated whole that relies on the interaction of each competency to achieve, in this case, Interprofessional collaboration. The capacity of learners or practitioners to demonstrate the integrated set of competencies and transfer their application into different contexts and into each situation is the measure of their ability to practice collaboratively. Hence, it is the resulting outcome and the associated judgment or assessment made in each specific situation that will determine the degree to which individual learners meet each Interprofessional competency domain and the framework as a whole. The judgment is based on the ability of the learner to integrate knowledge, skills, attitudes, and values, which comprise the measure of competence.

## Conclusion

The frameworks for IPC in Canada and Qatar provide the opportunity to use consistent language and concepts in education and practice. The competency framework provides a reference for Canadian education organizations and the Qatar Council for Health Care Practitioners (QCHP) to build explicit Interprofessional capabilities into their education offerings and accreditation requirements. The domains, competency statements, and descriptors represent a comprehensive picture of what it means to be a collaborative practitioner. The foundational concepts underpinning Interprofessional collaboration include the context of practice, the complexity of the issue, and the quality improvement approach to service delivery. All professional and allied professional workers can use the framework to assess where they fit along a continuum of competence in their practice.

The IPE framework models discussed serve several purposes across professions and allied professions. In the education context, the framework is effective to embed Interprofessional components of the curriculum in both academic and practice settings and can provide a foundation for assessment of competence in Interprofessional collaboration. Educators can use the framework as a guide to curriculum development and assessment of students. For practitioners it provides a guide for measuring one’s behavior, as well as that of others, in a collaborative practice environment. Regulators will be able to use the framework to enhance the regulatory standards of practice to include attention to Interprofessional practice. Accreditors of health education programs will be able to develop and implement Interprofessional education standards for assessing a program’s engagement with Interprofessional learning and practice.

The Qatar framework demonstrates how an effective and relevant competency framework can arise within a local and cultural context. Collaboration and research across different sectors such as post-secondary health care institutions and major hospitals in Qatar has led to the development of the Core IPE Health Competencies ( Johnson et al., 2015). An analysis of the College of Pharmacy’s framework, El-Awaisi et. al (2017) indicates that learning objectives for IPE initiatives need to be based on shared competency domains across several health professions and allied professions to provide a strong theoretical underpinning. There is still a lack of agreement for a single set of core competencies to assess IPE and competencies, but Qatar has a solid foundation for the application of IPE with the guidance of the developed framework. The set of Core IPE Health Competencies is useful for education, implementation in practice, and assessment of health care practitioners. It also provides an opportunity for application in continuing professional development.

While frameworks, whether in Canada, Qatar, or elsewhere in the world, will continue to evolve, they provide a broad strategy for creating a common understanding of Interprofessional collaboration that is applicable in several contexts. Health care educators will be able to use the proposed frameworks to design, implement, and assess Interprofessional education activities aimed at developing effective, collaborative practitioners.

## Take Home Messages

• Interprofessional Collaboration and Competency is increasingly required in education and practice.
• The Canadian Framework is flexible and valuable to a variety of learning environments and with students at different learning levels.
• The Qatar Framework incorporates elements of the Canadian Framework and is relevant to the Middle East region, and applicable in pre- and post-licensure settings.

## Notes On Contributors

Dr. Andrea Smilski is currently the Dean, Health Sciences for the College of North Atlantic, Qatar. Prior to this she served as a health and human services Educator, Associate Dean, and Dean in the Post-Secondary Education system in B.C. Canada. She has worked as a registered nurse, educator, manager, and leader in the health and education sectors in Canada as well as overseas in Jakarta, Indonesia. She has authored two manuscripts, edited two chapters in nursing education books, and authored and reviewed several peer reviewed articles in health journals. Dr. Smilski maintains an interdisciplinary research agenda that focuses on alternate health care models and organizational and employee health and wellbeing.

Mary Parrott is a health care educator who was instrumental in the set up the Respiratory Therapy Program in Qatar in 2005. As an educator, she has developed and delivered many CME/CPD activities with a focus on adult learning, and collaboration. As an experienced healthcare educator and accreditation specialist, she currently oversees the CPD program at College of the North Atlantic-Qatar in Doha, Qatar.
