# Supplementary material for: Interprofessional Competency Frameworks in Education
Source: MedEdPublish (2016). 2019 Mar 15;8:56. [Version 1] doi: 10.15694/mep.2019.000056.1 (PMC10712603; doi:10.15694/mep.2019.000056.1)
Supplement: Peer review history

# Reviewer report: Reviewer response for version 1

**DOI:** 10.21956/mep.19799.r29780

**Published:** 2019-05-08

**Authors:** Trevor Gibbs

**Affiliations:** 1 AMEE

This review has been migrated. The reviewer awarded 4 stars out of 5 An interesting paper written by authors ina unique situation. I enjoyed reading this paper and I particularly like the idea of whether competency frameworks travel well and how we should / might change these in relation to context and culture.A good read for those involved in new schools who wish to develop IPE- the frameworks- both- are very useful

Reviewer Expertise:

NA

No decision status is available

# Reviewer report: Reviewer response for version 1

**DOI:** 10.21956/mep.19799.r29782

**Published:** 2019-05-02

**Authors:** Hui Meng Er

**Affiliations:** 1 International Medical University

This review has been migrated. The reviewer awarded 4 stars out of 5 The interprofessional competency frameworks presented are useful as they clearly define the competencies for interprofessional collaborative practitioners based on literature evidences as well as stakeholders’ engagement. The two models are largely aligned and both take into consideration of the context, level of skill complexity and delivery methods, and are applicable to a wide range of health professions. The models are anchored on the ultimate objectives of improved patient care and health outcomes, placing interprofessional education across the continuum of learning via personalised approach. The frameworks were developed mainly based on the contexts and experiences in Canada and Qatar, but are potentially applicable to the elsewhere in the world. It would be useful to highlight the uniqueness (if any) of the healthcare systems in these countries with regards to interprofessional practice, so that readers can adapt the frameworks accordingly to their local contexts.

Reviewer Expertise:

NA

No decision status is available

# Reviewer report: Reviewer response for version 1

**DOI:** 10.21956/mep.19799.r29781

**Published:** 2019-03-18

**Authors:** Megan Anakin

**Affiliations:** 1 University of Otago

This review has been migrated. The reviewer awarded 4 stars out of 5 Thank you for this thought-provoking article that describes two interprofessional competency frameworks. I appreciate that this article emphasises that the construction and use of such frameworks provide us with the “opportunity to use consistent language and concepts in education and practice”. While we might dream about an overarching framework that can be used globally, the sociocultural nature of collaborative professional practice means that we must adapt frameworks for education and practice to our local and national contexts. Like the authors, I do not see a “lack of agreement for a single set of core competencies to assess IPE and competencies” as problematic. I appreciate that the authors outline a process for considering relevant aspects of educating and assessing interprofessional collaborative practitioners. This process will provide a helpful guideline for educators and researchers use as they develop interprofessional competency frameworks to address their particular needs.

Reviewer Expertise:

NA

No decision status is available
